# Factors influencing training transfer in nursing profession: a qualitative study

**DOI:** 10.1186/s12909-018-1149-7

**Published:** 2018-03-20

**Authors:** Fang Ma, Yangjing Bai, Yangjuan Bai, Weiguang Ma, Xiangyu Yang, Jiping Li

**Affiliations:** 1 Department of Nursing, West China Hospital, Sichuan University, Chengdu, China; 2 Department of Cardiovascular Surgery, West China Hospital, Sichuan University, Chengdu, China; 3 Department of Nursing, the First Affiliated Hospital of Kunming Medical University, Kunming, China; 4 School of Nursing, Peking Union Medical College, Beijing, China; 5 School of Nursing, Chengdu University of Traditional Chinese Medicine, Chengdu, China

**Keywords:** Training transfer, Training, Health professions, Qualitative research

## Abstract

**Background:**

There is a growing recognition that training is not translated into performance and the ‘transfer problem’ exists in organization training today. Although factors contributing to training transfer have been identified in business and industry, the factors influencing training transfer in nursing profession remain less clear.

**Methods:**

A qualitative descriptive study was undertaken in two tertiary referral hospitals in China from February 2013 to September 2013. Purposeful sampling of 24 nursing staffs were interviewed about the factors influencing training transfer.

**Results:**

Seven themes evolved from the analysis, categorized in 4 main domains, which described the factors influencing training transfer in nursing profession in trainee characteristics, training design, work environment and profession domain. The trainee characteristics domain included attitude and ability. The training design domain included training content and instruction method. The work environment domain included supports as facilitators and opposition as hindrance. The theme pertaining to the profession domain was professional development.

**Conclusions:**

Health care managers need to understand the factors influencing training transfer for maximizing the benefits of training. The right beliefs and values about training, the rigorous employee selection for training, the relevance of training content, training instructions facilitating learning and transfer, supports from peer, supervisors and the organization, organizational culture such as change, sharing, learning and support, and professional development are key to successful training transfer. Furthermore, managers should be aware of the opposition from co-workers and find ways to prevent it.

**Electronic supplementary material:**

The online version of this article (10.1186/s12909-018-1149-7) contains supplementary material, which is available to authorized users.

## Background

Training has long been held as the cornerstone of organizational development. Training in work organizations produces clear benefits for individuals, teams, organizations, and society [1, 2]. Effective training can facilitate the improvement of human capital [3], which means the promotion of nurses’ competency in nursing profession and links to the safety of patients and the ultimate goal of improving delivery of health care. On the contrary, a poorly trained workforce can lead to errors, injuries and even legal issues, all of which can be extremely costly [4].Within the ever-changing healthcare system, health care professionals have always been encouraged to update their knowledge and maintain clinical competence. Without a program of active learning no health care professional can hope to remain competent for more than a few years after graduation [5], which increases the pressure of nursing professionals to engage in continuous training and education. Successful training in health systems results in professionals’ enhanced knowledge and skills, improved staff satisfaction and retention, reduced patients’ mortality and quality patient care [6–8]. Due to the importance of training, much effort has been invested in this field with the expectation that training investment will lead to improvements in organizational performance and competitive advantage. Government and organizations spend billions of dollars every year on formal training and development programs [9], and vastly increasing investment in education and training of health workers has been reported globally [8, 10–13].

There is concern that the bulk of training expenditures do not transfer to the job. Estimates suggest that employees transfer less than 10% of training and development expenditures back to their workplace [14, 15]. It is reported in industry and health care settings as well that only a small amount of what learned in training are applied on the job [16–18].These findings confirm a serious transfer problem for organizations and indicate the glaring gap between training efforts and organizational outcomes. If organizations are to benefit from their training investments, it is crucial to improve the effectiveness of training and get the best use of training funds. It is suggested that the effectiveness of training depends on training transfer or transfer of training (TOT), which is defined as the extent to which knowledge, skills and attitudes learned in work-related training are applied on the job and subsequent maintenance of them over a certain period of time [19–21]. Although trainees may gain new knowledge and skills through training programs, learning alone is not sufficient for training to be considered effective. Of paramount importance is the positive training transfer, given that training transfer is considered to be the primary leverage point by which training influences organization-level outcomes and improved organizational performance can be obtained by facilitating training transfer [22, 23].To make training really work and the investments worthwhile, it is urgent to identify the factors that influence training transfer, thus ways can be identified to facilitate positive training transfer and to improve training effectiveness in the end.

Since training transfer problems have troubled organizations for a long time, some researchers have revealed the factors that influence training transfer, yet studies pertinent to training transfer in nursing profession are scarce. Baldwin and Ford presented a classic model of the transfer process which indicates training-inputs factors (trainee characteristics, training design, and work environment) are posited to have direct and indirect effects on the conditions of transfer [20] (See Appendix). Trainee characteristics consist of the individual’s ability, motivation, and personality factors. Training design factors are principle of learning, sequencing and training content. Work environment characteristics include support and opportunity to use [20].Since then, subsequent research on this theme continued to fall within the three broad categories of trainee characteristics, training design, and work environment factors. As for trainee characteristics aspect, cognitive ability, self-efficacy, motivation, personality, perceived utility, career/job variables and locus of control have been identified to affect training transfer [15, 18, 21, 24–26].In training design area, factors such as content relevance, error management, behavioral modeling, and technological support were reported to have effects on training transfer [15, 27–30]. In the work environment area, transfer climate, supervisor/peer support, opportunity to perform, accountability, and follow-up have been identified to affect the TOT [15, 21, 24, 31–33]. Despite a growing body of variables have been identified on this topic, whether they are applicable to the nursing profession remains questionable. This may in part be attributed to the fact that many of these researches were conducted in business and industry, and related researches in health sectors are scarce. Moreover, due to the heterogeneity of setting, sample and population of the studies, the research findings are inconsistent and conflicting [2, 4, 21]. These findings alert us that previous identified variables may not be applicable for studying the training transfer process in nursing profession. In this qualitative study, the purpose was to explore nursing professionals’ perspectives on the factors that influence training transfer, which will aid the understanding of the transfer process in nursing profession and gain insight into the way to improve training transfer.

## Methods

### Study design

A qualitative descriptive study using in-depth, face-to-face interviews was adopted in this study. The study was approved by the relevant regional ethics committee. Participants were given written information about the study’s aims and procedures, and about their right to withdraw at any time. Participants were assured that their names would not be used and that confidentiality would be maintained by the researchers. Before data collection informed consent was obtained from each participant. Participation was voluntary.

### Participants and setting

Participants were nursing staffs and administrators at two tertiary referral hospitals in two provinces respectively. There are geographical, cultural and economic differences between the two sites. One institution is located in the border area of China with many ethnic minority groups living there and the economy is less developed, the other one is in the inland area of China and the economy is more developed. The differences may result in different institutional cultures and provide a chance to broaden our perspectives on the research topic. To obtain as heterogeneous sample as possible and to achieve maximum variation across a range of participants’ backgrounds, the purposive sampling was adopted. Nurses who have experienced training as trainers or trainees in the health care setting may provide rich and detailed information. Nurse managers involved in training management may also provide related information. Nurses with experiences of different types of training and nurse managers in charge of training in clinical settings were selected in this study. Some participants were considered by their supervisors to be typical of conducting positive training transfer in health care settings, and some were considered to fail to transfer; hence the sample was considered representative of the study population. Sampling continued concurrently with data analysis until no new themes were identified.

### Data collection

Nurses and nurse managers participated in semi-structured interviews between February 2013 and September 2013. Each interview was audio-taped and the average time for each interview was approximately half an hour. The following topics guided the interviews: (1) Recall your training experiences home and abroad, did you apply the new knowledge and skills learned in training on your work environment and what supported or hindered you? (2) Considering your training experiences as trainers, trainees, and training managers, what factors facilitate and hamper the application of what learned in the training on the workplace. An additional file shows this in more detail (see Additional file 1). Researcher (MF) with experiences in qualitative research methods conducted all the interviews in Chinese. The researcher had no supervisory relationship to the participants and they were assured that their responses would not affect their performance appraisal. Participants were asked to verify the accuracy of the information discussed during the interview before the end of the interview. After being recorded, the quotes were transcribed verbatim and translated from Chinese to English by two translators.

### Analysis

Data analysis was carried out concurrently with data collection until the research team determined that no new themes were emerging. The data were analyzed using qualitative content analysis [34, 35].The model of the transfer process presented by Baldwin and Ford was used to guide the analysis [20], which could inform the initial direction of qualitative data analysis without limiting the identification of new themes. All tape-recorded interviews were transcribed verbatim immediately after the interview. The process of analysis included repeated listening to the audiotape, and thoughtful reading and rereading of the transcribed texts. Phrases within the transcripts that pertained to participants’ views of the enablers and inhibitors of training transfer were highlighted. Data were read using line-by-line analysis to identify codes appearing to capture key thoughts or concepts of the interview discussion. The various codes were compared based on differences and similarities and sorted into categories and themes. To eliminate the risk for bias, two researchers (MF and BY1) with expertise in qualitative data analysis analyzed the data independently. In the cases of differences in the analysis, the two researchers communicated with each other to assure that an inductive process occurred and that was consistent with the views of the participants of the study. Agreement was reached through discussion where differences in analysis appeared.

### Ethical considerations

The study was approved by the ethics committees of the university. Participants were assured that their names would not be used and confidentiality would be maintained by the researchers. Before data collection written informed consent was obtained from each participant.

## Results

The sample included 21 nurses and 3 nurse managers in charge of training with 20 from one hospital and 4 from another one. Participants included 21 female and 3 male medical staffs, and represented 8 master degree, 14 bachelor degree, and 2 diploma degree professionals. Content analysis of the qualitative data resulted in the identification of seven themes and they have been grouped into four domains. Domain A: Trainee characteristics; Domain B: Training design; Domain C: Work environment; Domain D: Profession. The themes were verbally described; examples are given verbatim.

### Domain a: Trainee characteristics

#### Theme 1: Attitude

Participants mentioned that the attitude of trainees toward whether or not using the training content in the practice affected training transfer. As we identified in this study, some trainees did not want to apply what they learned in training for the purpose of monopoly and competitiveness edge, hence hindered the TOT:

*“I once sent a nurse to learn the skills of tracheotomy care from another hospital, she told me there were nothing new, but later I knew she lied and I asked why.…She didn’t want to utilize the skills and knowledge learned in training setting to the workplace. She was afraid that after application, her advantage and competitiveness gained from training would disappear, she wanted to apply the skills someday to help her get promotion.”(Participant 10)*

Besides, some participants indicated that individuals’ attitude toward training influenced their judgment about the purpose of training, which impacted the application of the training content. Especially the wrong attitude toward training impeded training transfer:

*“Some colleagues in my department consider that training is a kind of employee benefits, or means of providing financial incentives, or meeting the training quota. Therefore, it is a good chance to get relief from work, they can choose to learn or not as they like… Usually, they enjoy their leisure time during the training period… There is little possibility to apply the training content in the workplace.”(Participant 1)*

#### Theme 2: Ability

Participants proposed that whether the trainee had the ability to grasp and understand what was learned in the training, whether the trainee had time, energy and ability to apply the training content on the workplace affected the transfer process, therefore the basis of staff selection for training is important:

*“Sometimes internal rotation or turn is used as the principle of staff selection for training in our institution, often the right person is not selected in the training program. If the trainee could not understand what was taught or had no ability to utilize the learned skills and knowledge in the workplace, nothing would happen……”(Participant 6)*

It was reflected in the interviews that the self-efficacy of trainees influenced training transfer. When the trainees felt confident and self-assured about applying learned abilities on their jobs and could conquer the obstacles that hindered the use of leaned knowledge and skills, it was possible that positive transfer might happen. As one participant stated her experience:

*“I applied the problem-based learning in the clinical teaching after I learned related knowledge and skills, but I found the students did not cooperate …I was confident that I could overcome the difficulties. I asked for help from my teachers and did more work to get cooperation from the students, I really did…”(Participant 16)*

### Domain B: Training design

#### Theme 1: Training content

All participants mentioned that whether the training content matched their work requirement, reflected the needs of the trainees, their jobs and their departments, and helped them resolve the problems that affected the TOT. Some nurses addressed their concerns about the unsuitable training content to the culture and policy, as a typical example like this:

*“The quality improvement program I leaned in Thailand was great, but it could not be applied in our department now because of different culture and policy.”(Participant 1)*

#### Theme 2: Instruction method

Besides the training content, how the training was conducted also influenced trainees’ grasp of trained knowledge and the TOT. Participants mentioned that whether the instruction method could facilitate trainees’ understanding and mastery of the content was key to training transfer, and they pointed out that the training design should be practice-oriented:

*“The most effective instruction method is active learning through behaviouristic methods, such as simulation and role play, which enable us to learn and apply.”(Participant 3)*

### Domain C: Work environment

#### Theme 1: Supports as facilitators

Participants considered the supports from the organization, supervisors and co-workers acting as facilitators in the work environment, which contributed to the positive training transfer. Organizational supports were cited by the participants as: the organizational policy demonstrated the positive link between utilization of the training content and reward of the trainees; opportunities to use; adequate materials, financial and human resources; the coordination between departments; and organizational culture benefiting training transfer. One participant stated her confusion and difficulty in her application of the learning content:

*“The hospital information system should be improved, or we could not collect the data necessary for applying performance appraisal which was learned in the training program.”(Participant 11)*

Participants mentioned the culture of change, sharing, learning and support in the organization was conducive to training transfer. In some cases, participants reported that in an organization where work group were open to change and willing to invest energy to change, the TOT could be facilitated. On the contrary, resistance to change impeded the TOT:

*“Why was the IV indwelling needle hard to be applied in our department though we were trained many times for its application? Because in this resistance to change culture, my colleagues were costumed to using the disposable IV infusion set needle and did not want to change……”(Participant 5)*

Sharing culture was perceived as an important enabler to the successful training transfer:

*“In a sharing culture, we share with each other what we learned, our successful or unsuccessful experiences in workplace, and more people grasp the training content, which facilitates the application.”(Participant 2)*

When talked about supervisor supports, participants mentioned the supports included: motivating and guiding the utilization of learned knowledge and skills; providing different resources and opportunities to apply what learned in training; inspecting and providing feedback about the trainees’ application of the training content. Moreover, they proposed that instead of just verbal support from their supervisors, their working together with the trainees to overcome the obstacles in the transfer process facilitated training transfer:

*“After I was trained for the knowledge and skills of chronic wound care in a training program, my supervisor set up a chronic wound care clinic and asked me to work there. She often came to the clinic to see what should be improved. I considered that my supervisor was the person I could count on and whenever I encountered difficulties, she motivated me and worked with me to resolve them… which promoted training transfer.”(Participant 12)*

Participants stated that the supports from their colleagues, such as verbal encouragement and working with the trainees on problems encountered while applying the training content could facilitate the TOT:

*“After I learned something new in the dialysis technique, I needed the supports from doctors and nurses to use the skills. They gave me feedback, trusted me, supported me, and helped me resolve the problems encountered during the application of the new skills, then we got a successful training transfer.”(Participant 20)*

#### Theme 2: Opposition as hindrance

The interviewees highlighted that one of the obstacles in the process of training transfer was opposition from their co-workers, such as conflict in point of view between the trainees and their colleagues, resisting or opposing the use of learned skills and knowledge by colleagues in the application process.

*“During my application of the evidence-based nursing practice after the training program, some of my colleagues thought it cost too much time and energy, and some considered there was a risk because we had never done it before…what I got were verbal cynicism and opposition.…….. I gave up.”(Participant 16)*

### Domain D: Profession

#### Theme 1: Professional development

Participants indicated that due to the limitation of the professional development, they considered no matter how much effort they exerted in the learning and application of training content, it was hard to get reward or achieve self-development. They were confused in learning and utilizing the training content, which hindered the TOT.

*“The senior and junior nurses almost do the same repetitive and routine work, and our professional development is limited. Nurses thought it useless to learn from the training… We should deepen and broaden our scope of practice to promote our professional development, which can also justify the learning and application in the training process.” (Participant 13)*

## Discussion

This study offers a view of the factors that influence training transfer from the perspectives of nursing staff in China. Compared with other researches, some similarities and differences occurred. Individual’s attitude toward training such as motivation to transfer have been found to influence training transfer in other researches [2, 20, 36]. Our study suggests that the underlying cause of motivation to transfer might involve knowledge sharing. Ardichvili [37] pointed out that today’s economic conditions in China were extremely competitive, and a widely accepted proverb was “knowledge is power”. When people acquired new knowledge, they believed that it was the key to their success and were likely to guard it instead of sharing it, and they considered that reservation instead of application was the best way to guard the new knowledge, which inhibited the TOT. As an old Chinese saying goes that ‘the catholic mentor was starved by his disciples’, which indicates knowledge power is critical and individuals are unwilling to share their knowledge and experiences with others [38]. In Chinese culture, face saving has a negative effect on the intention to share knowledge [38], and the team members in health care settings may not acquire knowledge from their colleagues to avoid the embarrassment it might cause. Furthermore, the hierarchical structure in hospital and the medical culture of shame, blame and silence have been suggested to hinder knowledge sharing [39]. All above suggest that individual level and organizational level factors hinder knowledge sharing in Chinese health care settings, which may block the TOT. Hence, knowledge sharing should be continuously promoted and barriers should be overcome. Strategies such as management support, and linking knowledge sharing with rewards and performance appraisal are strongly recommended. Moreover, health care settings should create opportunities for health professionals interactions to occur and professionals’ rank, position in the hospital hierarchy, and seniority should be deemphasized to facilitate knowledge sharing and the TOT in the end [40]. Our results alert us that the attitude toward the purpose of training held by health staff are likely to influence the TOT, hence the right beliefs and values of training should be developed in health care professions. As Subedi [41] suggested we should discourage the tendency to take training as a means of financial gains or relief from work for better training results. Performance improvement and achieving organizational goals should be perceived as of the highest value for training.

Individual ability and self-efficacy might influence training transfer, which have been reported in other research findings [2, 20, 36]. Of particular note, our findings showed that personal ability influenced training transfer, which reminds us to avoid bias in staff selection for training to ensure that the selected trainees have the ability to grasp the training content and transfer what have been learned to the workplace [30].The selection of health professionals to be sent for training should be affected by formal criteria and procedure such as organizational needs and job or performance requirements. Practices of employee selection for training based on internal rotation or turn indicates a situation not especially conducive to the TOT [41].

Our results identified that in training domain, training content and instruction method had effects on training transfer. It has been evidenced in other studies that relevance and suitability of training content influenced training transfer [18, 30, 36].The results reinforce that we should design and deliver training that is practical and responsive to real workplace. Besides, the traditional didactic approach is not suitable for adult learning, and there is no one-size-fits-all approach towards all kinds of training [42]. According to different training content and trainees, diverse types of instruction methods are needed to generate effective learning and allow the learned knowledge and skills to be effectively transferred [42, 43].

Factors that influence the TOT in the work environment have been identified in the qualitative analysis. As participants stated, the organizational supports such as reward systems emphasizing the rewards and incentives for the TOT, providence of the resources necessary for trainees to use the learned knowledge and skills were beneficial to the successful training transfer in our study, which were also reported in other researches [31, 44]. Our findings suggest that supervisor supports such as requiring the trainees to use their training and rewarding them, giving post-training follow-up, allocating opportunity/time for the trainees to implement learning, helping trainees overcome obstacles encountered in the transfer process have influence on the TOT, besides, peer supports were cited as facilitator to training transfer, which have also been evidenced in other researches [30, 36]. In our study, organizational culture such as change, sharing, learning and support might influence training transfer, which have been supported in other researches [2, 36, 44–46]. Of particular note, participants in our study indicated that sharing culture could influence training transfer, which might be associated with personal motivation to transfer and has been discussed above. It is noteworthy that sharing culture should be stressed in health care settings.

The negative role of opposition from co-workers in training transfer was identified in our study, which has not drawn enough attention among health care professionals. As participants suggested that in the transfer process, they might receive cynicism, ridicule, non-cooperation, opposition or hostile behavior from their colleagues. These hostile and disruptive behaviors undermined trainees’ motivation to apply their learning into practice and had negative effect on the trainees, such as anxiety, poor morale and even avoidance at work [47], ultimately hindered the TOT [15].The reasons for opposition from co-workers might be as follows: Firstly, the selection process for training might be largely affected by informal criteria [41] and the training investment is unfair among individuals, which might lead to conflicts among colleagues and subsequent opposition from co-workers in the application of the learned knowledge and skills; secondly, the application of training content might involve colleagues’ effort and improve their workload, which make them resist applying the training content; thirdly, due to fear of the uncertain outcomes accompanied by the utilization of new skills, the colleagues resist the application of newly learned skills as a result of unwillingness to change and give up their comfort zone [48]. Hence, rigorous staff selection for training, organizational culture such as support and change are important for the avoidance of opposition from co-workers and can facilitate positive training transfer.

Professional development is not mentioned in Baldwin and Ford’s model of transfer. Nowadays nursing is seen as having limited career opportunities as well as restricted autonomy, which result in limited professional development [49, 50]. The limited professional development may lead to the development of a negative professional identity and career commitment, which may decrease individuals’ motivation to learn and reactions to training [24], hence hinder the TOT. It is extremely important to develop nurses’ opportunities for clinical expertise and expand their authority and responsibility. These developments will help improving nurses’ professional development to establish a public image and professional identity that recognizes the value of their professional and educational development [49], which can facilitate the TOT.

In our analysis, we found that participants from the hospital in the more developed economy area stated more examples of positive training transfer. On the contrary, experiences of negative training transfer were mentioned mostly by participants from the less developed economy area and the theme “professional development” was suggested by them. The reason might be that developed economy contributes to more supports from the organization such as financial and material resources. Furthermore, different cultural and geographical characteristics might influence institutional culture, which may impact training transfer. We consider it wise to included diverse institutions in our research to provide the chance for us to contact with participants from different cultural backgrounds with various experiences in work and training, which can help us get enriched information concerning our research topic.

### Limitations

The limitations of the present study refer to its inclusion of nurses from two hospitals in China, and this may not be representative of all nurses in China. A relevant follow up study might be to use the same design with professionals from other countries to validate the international nature of the study. In this research, the model of the transfer process presented by Baldwin and Ford was used to guide the analysis. Although we tried to use this model to inform the initial direction of qualitative data analysis without limiting the identification of new themes, we might be constrained by this model in the analysis process. More models related to training transfer such as Holton’s Model and Noe’s Model may be considered in the future study.

## Conclusions

Our findings illustrate that the factors influencing training transfer in nursing profession is complex, which are coincident with other research findings that due to the complexity and dynamics of the training transfer process, it should be understood and fostered by examining a relatively complete system of influences. Exploring nurses’ perspectives on the factors that influence training transfer can inform efforts to health care managers in how to achieve positive training transfer. The results of this study illuminate to health care managers how important it is that the right beliefs and values of training be clarified, and a rigorous selection process for training be appropriately considered for successful training transfer. Our research highlights that knowledge sharing influences training transfer and more strategies should be identified to facilitate knowledge sharing and training transfer in the end. It is without any doubt that job-related and need-based training is desirable for higher training transfer, besides, training instructions such as simulation and role-play increase the likelihood of positive training transfer. Organizational policy that links performance appraisal and reward systems to training transfer should be emphasized in health care management. In addition, allocating opportunity, time and resources for the trainees to implement learning by the the organization and supervisors; goal setting, guidance, inspection and feedback in the application process by supervisors; cooperation and support from co-workers facilitate training transfer. The negative influence of opposition from co-workers should not be ignored and further researches are needed to explore this variable. Most importantly of all, we should create a positive organizational culture that promotes learning, change, support and sharing in the organization to facilitate training transfer. Furthermore, efforts should be invested to improve the professional development of nursing.

### Additional file

Additional file 1: Interview Guide. The interview guides developed for the study. (DOCX 15 kb)

## Figures and Tables

**Fig. 1:**
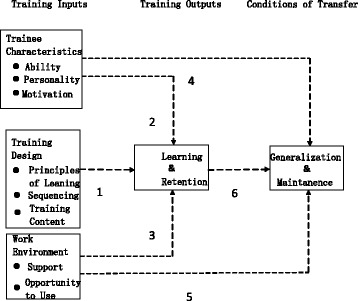
A Model of the Transfer Process [20]

## Abbreviation

TOT: Transfer of Training
