# Supplementary material for: Factors influencing training transfer in nursing profession: a qualitative study
Source: BMC Med Educ. 2018 Mar 20;18:44. doi: 10.1186/s12909-018-1149-7 (PMC5859543; doi:10.1186/s12909-018-1149-7)
Supplement: Supplementary file 1 — Interview Guide. The interview guides developed for the study. (DOCX 15 kb) [file 12909_2018_1149_MOESM1_ESM.docx]

Interview Guide

1. Please talk about your impressive training experiences in your working life. For example, the time, location, training content and your perception about these training experiences.
2. Would you please talk about your successful training experiences and why do you think those trainings are successful. What did you benefit from those successful training experiences and how about your organization?
3. Would you please talk about your unsuccessful training experiences and why do you think those training are unsuccessful. What did you lose from those unsuccessful training experiences and how about your organization?
4. Recall your training experiences home and abroad, did you apply the new knowledge and skills learned in training on your work environment and what supported or hindered you?
5. Considering your training experiences as trainers, trainees, and training managers, what factors facilitate and hamper the application of what learned in the training on the workplace.

Thank you for your participation, if you have any questions or anything to add about the interview, please do not hesitate to contact me at any time.
